# Proximal femoral replacement using the direct anterior approach to the hip

**DOI:** 10.1007/s00064-022-00770-x

**Published:** 2022-05-31

**Authors:** Martin Thaler, Theodore T. Manson, Boris Michael Holzapfel, Joseph Moskal

**Affiliations:** 1 Arthroplasty Center, Helios Klinikum Munich West, Steinerweg 5, 81241 Munich, Germany; 2 Universitätsklinik für Orthopädie, Medizinische Universität Innsbruck, Anichstraße 35, 6020 Innsbruck, Austria; 3 Center of Orthopaedics, Trauma Surgery and Rehabilitation Medicine, University of Greifswald, Greifswald, Germany; 4 Department of Orthopaedic Surgery, University of Maryland, 21204 Baltimore, MD USA; 5 Department of Orthopedics and Trauma Surgery, Musculoskeletal University Center Munich (MUM), University Hospital, LMU Munich, Munich, Germany; 6 Department of Orthopedic Surgery, Virginia Tech Carilion School of Medicine, Institute for Orthopedics and Neurosciences, 24014 Roanoke, VA USA

**Keywords:** Revision total hip arthroplasty, Massive bone loss, Femoral revision, Sarcoma, Metastatic bone disease, Revisionsendoprothetik, Massiver Knochenverlust, Femorale Revision, Sarkom, Knochenmetastase

## Abstract

**Objective:**

Proximal femoral replacement (PFR) is a salvage procedure originally developed for reconstruction after resection of sarcomas and metastatic cancer. These techniques can also be adapted for the treatment of non-oncologic reconstruction for cases involving massive proximal bone loss. The direct anterior approach (DAA) is readily utilized for revision total hip arthroplasty (THA), but there have been few reports of its use for proximal femoral replacement.

**Indications:**

Aseptic, septic femoral implant loosening, periprosthetic femoral fracture, oncologic lesions of the proximal femur. The most common indication for non-oncologic proximal femoral placement is a severe femoral defect Paprosky IIIB or IV.

**Contraindications:**

Infection.

**Surgical technique:**

In contrast to conventional DAA approaches and extensions, we recommend starting the approach 3 cm lateral to the anterior superior iliac spine and performing a straight incision directed towards the fibular head. After identification and incision of the tensor fasciae lata proximally and the lateral mobilization of the iliotibial tract distally, the vastus lateralis muscle can be retracted medially as far as needed. Special care should be taken to avoid injuries to the branches of the femoral nerve innervating the vastus lateralis muscle. If required, the distal extension of the DAA can continue all the way to the knee to allow implantation of a total femoral replacement. The level of the femoral resection is detected with an x‑ray. In accordance with preoperative planning, the proximal femur is resected. Ream and broach the distal femoral fragment to the femoral canal. With trial implants in place, leg length, anteversion of the implant and hip stability are evaluated. It is crucial to provide robust reattachment of the abductor muscles to the PFR prosthesis. Mesh reinforcement can be used to reinforce the muscular attachment if necessary.

**Postoperative management:**

We typically use no hip precautions other than to limit combined external rotation and extension for 6 weeks. In most cases, full weight bearing is possible after surgery.

**Results:**

A PFR was performed in 16 patients (mean age: 55.1 years; range 17–84 years) using an extension of the DAA. The indication was primary bone sarcoma in 7 patients, metastatic lesion in 6 patients and massive periprosthetic femoral bone loss in 3 patients. Complications related to the surgery occurred in 2 patients (both were dislocation). Overall, 1 patient required reoperation and 1 patient died because of his disease. Mean follow-up was 34.5 months.

## Abstract (de)

**Operationsziel:**

Die proximale Femurersatzprothese ist oft eine Rettungsprozedur in der Endoprothetik. Ursprünglich wurde diese Technik für Rekonstruktionen nach Sarkom- oder Metastasenresektion verwendet. Diese Techniken können allerdings auch für nichtonkologische Rekonstruktionen verwendet werden in Fällen mit massivem Knochenverlust im Bereich des proximalen Femurs. Der direkte vordere Zugang (DAA) wird regelmäßig für die Revisionsendoprothetik der Hüfte verwendet, aber es gibt nur wenige Berichte über die Technik des proximalen Femurersatzes über diesen Zugang.

**Indikationen:**

Aseptische Femurimplantatlockerung, periprothetische Femurfraktur, Sarkom im Bereich des proximalen Femurs, Metastase im Bereich des proximalen Femurs. Die häufigste nichtonkologische Indikation ist ein femoraler Defekt nach Paprosky IIIB oder IV.

**Kontraindikationen:**

Infektionen.

**Operationstechnik:**

Im Gegensatz zum Standard-DAA-Zugang und zu Erweiterungen empfehlen wir bei der Implantation einer proximalen Femurersatzprothese einen geraden Hautschnitt, beginnend 3 cm lateral der Spina iliaca anterior superior in Richtung des Fibulaköpfchens. Nach Identifikation und Inzision der Faszie des M. tensor fasciae latae proximal und Mobilisation des Tractus iliotibialis nach lateral distal, kann der M. vastus lateralis nach medial weggehalten werden. Um Verletzungen der Äste des N. femoralis zu vermeiden, die den M. vastus lateralis innervieren, sollte bei der Präparation besonders darauf geachtet werden. Nach distal kann der DAA-Zugang bis zum Kniegelenk erweitert werden, so dass sogar die Implantation einer totalen Femurprothese möglich ist. Die Resektionshöhe sollte nun bestimmt, mittels einer Röntgenaufnahme kontrolliert und mit der präoperativen Planung abgeglichen werden. Mit Hilfe von Probeimplantaten können nun die Beinlänge, die Anteversion des Femurs und die Luxationswahrscheinlichkeit überprüft werden. Es ist unbedingt notwendig, die Abduktoren suffizient am proximalen Femur zu refixieren. Hierzu kann ein Anbindungsschlauch verwendet werden.

**Weiterbehandlung:**

Bewegungseinschränkungen bezüglich der Außenrotation und Extension für 6 Wochen. In den meisten Fällen ist Vollbelastung nach der Operation möglich.

**Ergebnisse:**

Die Implantation einer proximalen Femurersatzprothese wurde in 16 Patienten (Durchschnittsalter: 55,1 Jahre; Min. 17 Jahre; Max. 84 Jahre) mit einer Erweiterung des DAA durchgeführt. Indikation für die Operation war bei 7 Patienten ein primäres Knochensarkom, bei 6 Patienten eine metastatische Läsion des proximalen Femurs und bei 3 Patienten ein massiver periprothetischer Knochenverlust im Bereich des proximalen Femurs. Operationsbezogene Komplikationen ereigneten sich bei 2 Patienten (Luxation). Ein Patient verstarb aufgrund seiner Grundkrankheit (Sarkom). Die durchschnittliche Nachuntersuchungszeit betrug 34,5 Monate.

## Introductory remarks

Proximal femoral replacement (PFR) is a salvage procedure originally developed for the treatment of bone tumors and metastatic cancer. These techniques were adapted for the treatment of non-oncologic reconstruction for cases involving massive proximal bone loss [1, 3, 5, 8, 11, 12, 20].

The direct anterior approach (DAA) is readily utilized for revision total hip arthroplasty (THA) [2, 4, 6, 7, 9, 10, 13–18], but there have not been many descriptions of its use for proximal femoral replacement. The goal of proximal femoral replacement is to reconstruct massive defects of the proximal femoral bone due to osteolysis or after resection of primary or secondary bone tumors. The most common indication for non-oncologic proximal femoral placement is severe Paprosky IIIB or IV femoral defects [19]. Non-oncological cases with a missing greater trochanter are excellent candidates for a PFR reconstruction with the DAA. The lateral approach is a widely spread surgical technique for PFR. However, we prefer to use the direct anterior approach to the hip for these procedures as it allows for supine positioning, direct assessment of acetabular component position using fluoroscopy, and direct comparison of leg length intraoperatively. In addition, the primary skin incision can be used in a revision scenario and DAA trained surgeons can use their favorite approach for revisions and oncologic procedures. In particular cases, the vastogluteal sling can be protected and less muscular transection is needed. Disadvantages of PFR with the DAA are that knowledge about distal and proximal extension of the DAA is needed, and the surgeon should be well out of their learning curve with the DAA. Proximal femoral replacement through the direct anterior approach is a straightforward technique for management of massive proximal femoral bone loss. With the patient supine, anesthesia access and pulmonary oxygenation is improved which can be useful in the complex patients who are usually undergoing this procedure. Leg lengths can be compared directly and fluoroscopic assessment of acetabular component position is straightforward.

In addition, implants that allow for either dual mobility or constrained liners should be utilized, as dislocation is the most common complication of proximal femoral replacement regardless of the approach used [7, 17].

With careful preoperative work-up to rule out infection and careful anatomic dissection during surgery, the direct anterior approach can be a reliable and consistent surgical technique for proximal femoral replacement.

## Surgical principle and objective

Proximal femoral replacement revisions can be performed with any surgical approach to the hip joint, like the posterior or the lateral approach. The femur can be approached with either a proximal or a distal extension or a combination of both. The objective is to reconstruct a massive defect of the proximal femur due to bone loss or tumor resection in order to mobilize the patient as early as possible.

## Advantages

- Supine positioning (easy application of intraoperative fluoroscopic control)
- Easy assessment of acetabular component position
- Easy intraoperative assessment of leg length
- Anesthesia access and pulmonary oxygenation is improved in complex patients
- DAA trained surgeons can use their favorite approach for revisions.
- If there is no tumor involvement in the greater trochanter and the resection margins allow it, the greater trochanter or a small bony attachment can be osteotomized from the femur and therefore the attachment of the abductors, the gluteal muscle can be preserved.

## Disadvantages

- Surgeon experience with the direct anterior approach to the hip and the distal extensile extension of this approach are mandatory for this surgical technique.
- Lateral femoral cutaneous nerve lesions are possible: meralgia, hypesthesia.
- Perforating arteries and veins on the lateral–posterior aspect of the femur can bleed profusely.
- Care must be taken to avoid damage to the branches of the femoral nerve innervating the vastus muscle.

## Indications

- Bone tumors of the proximal femur
- Metastatic lesions of the proximal femur
- Massive bone loss of the proximal femur (Paprosky IIIB or IV femoral defects)

## Contraindications

- Infection
- In oncological cases: If a resection with wide surgical margins cannot be performed

## Patient information

- General surgical risks, e.g., thrombosis, infection, wound healing problems, postoperative hemorrhage
- Higher risk of dislocation and infection as well as limp with these complex procedures
- Recalcitrant infections of a proximal femoral replacement can result in hip disarticulation
- Especially in patients in poor general condition the potential results and concerns associated with this procedure must be discussed with the patient preoperatively
- Tumor recurrence
- Metastasis in cases of malignant bone tumors
- Injury of the lateral femoral cutaneous nerve occurs more frequently with the anterior approach for PFR. Burning sensations, hypoesthesia and meralgia paresthetica might result.

## Preoperative work up

**Fig. 1:**
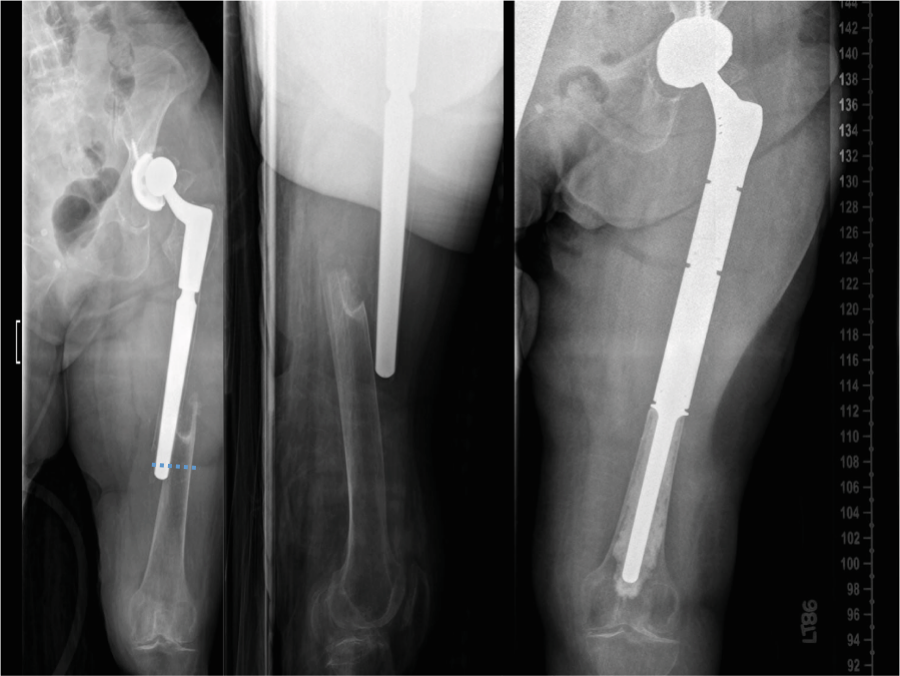
X‑ray showing a complex periprosthetic fracture with little proximal bone stock remaining. The patient was treated with proximal femoral replacement and acetabular component revision. Cemented fixation was used due to osteoporosis

- In non-oncological cases all patients have a preoperative hip aspiration to rule out infection
- Regular oncological work up
- Magnetic resonance imaging and computer tomography not older than 4 weeks in oncologic cases to evaluate soft tissue involvement of the tumor
- Templating of the resection level (Fig. 1)
- The biopsy of a suspected bone sarcoma should be carefully planned according to the site of the definitive surgery (anterior approach)
- Multidisciplinary approach that includes orthopedic, medical and radiation oncologists, plastic surgeons, pathologists, as well as radiologists with expertise in bone tumors

## Instruments and implants

- Dual mobility and constrained acetabular liners for the acetabulum should be available in complex cases.
- A bipolar head may be used in select oncologic cases without acetabular reconstruction.
- Modular implants are highly recommended to provide adequate reconstruction of leg length during surgery.

## Anesthesia and positioning

- General anesthesia is recommended due to the estimated surgical time.
- Antibiotic prophylaxis: Cefazolin or cefuroxime; vancomycin if confirmed penicillin allergy (additional dose if operating time exceeds 2–4 h (6–12 h for vancomycin) or there is “significant” blood loss).
- The use of tranexamic acid to reduce blood loss and to reduce the risk of transfusion might be considered.
- The patient is positioned supine on a flat radiolucent table.
- We do not use Hana or fracture table for this procedure as they actually hinder the femoral preparation and the direct comparison of leg length.

## Surgical technique

Figures 2, 3, 4, 5, 6, 7, 8, 9, 10, 11, 12.

**Fig. 2:**
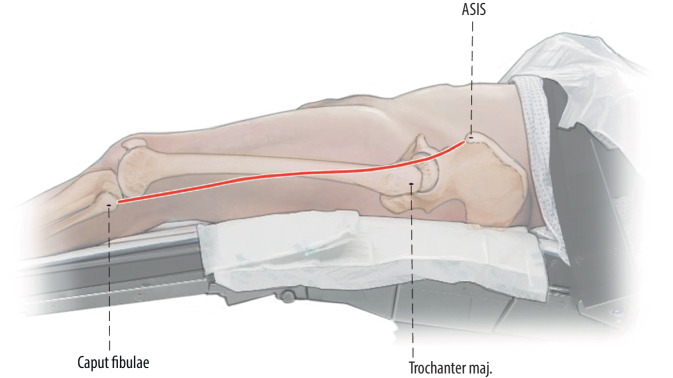
A direct extension of the anterior hip incision [16] is used. Rather than complex curvilinear incisions, we prefer a straight incision starting 1 cm lateral to the anterior superior iliac spine (ASIS) directed towards the fibular head

**Fig. 3:**
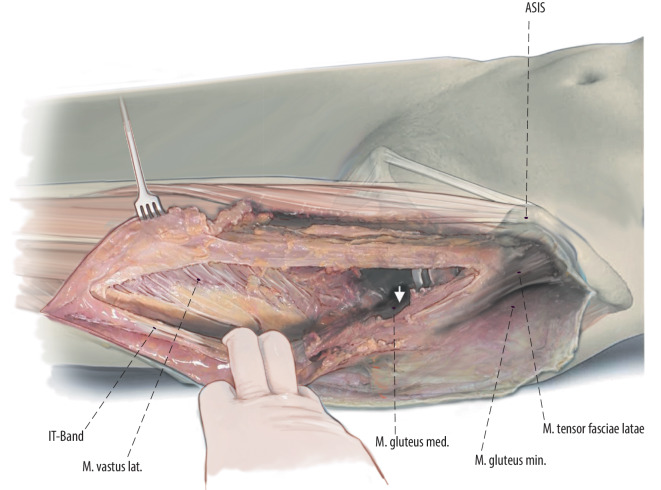
The anterior approach of the hip is extended distally. Then the thin fascia between anterior border of the tensor fascie latae (TFL) and the quadriceps muscle is split longitudinally as far as distally needed. Then the fascia, TFL muscle and iliotibial band can be bluntly mobilized from the underlying vastus lateralis muscle to lateral. The vastogluteal sling should be protected if possible to provide better functional results after the surgery. After exposure of the hip joint itself and the acetabulum, the surgery is proceeded with subvastus exposure of the lateral femur or lateral implant (right hip)

**Fig. 4:**
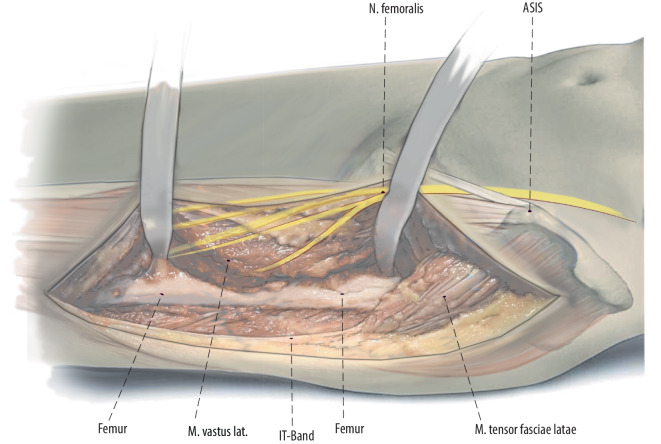
The surgery is proceeded with subvastus exposure of the lateral femur or implant. The femoral shaft is exposed in a lateral to medial and posterior to anterior direction to avoid damage to branches of the femoral nerve [10]. It is crucial for oncological as well as non-oncological resections to define the level of exposure and resection prior to the surgery. In oncological cases the soft tissue involvement of the tumor has to be respected and included in the resection. After exposure of the femur, we release the vastus lateralis from the intertrochanteric line on the anterior aspect of the femur

**Fig. 5:**
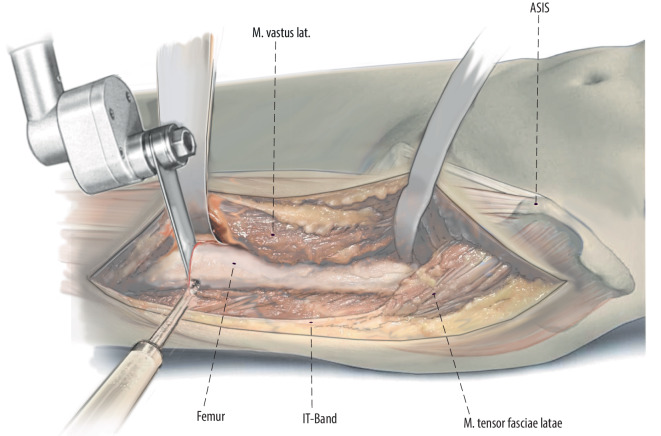
To remove the proximal femur, the surgeon can either start proximally and work distally or start distally and work proximally. Resect the femoral shaft according to the preoperative template at an area of robust bone that allows for stable fixation. The femur is resected at the templated location using a saw. A posterior wide Cobb elevator and medial Cobra retractor protect the soft tissues and neurovascular structures

**Fig. 6:**
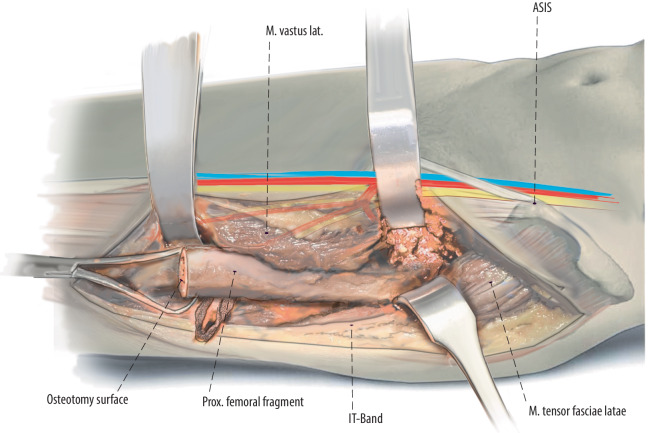
Then the distal aspect of the fragment is grasped to be resected by using subperiosteal elevation and careful Bovie electrocautery to remove the proximal femoral fragment. In oncological cases, resection margins regarding soft tissue involvement have to be respected. After removal of the proximal femoral segment, the acetabulum can be approached easily

**Fig. 7:**
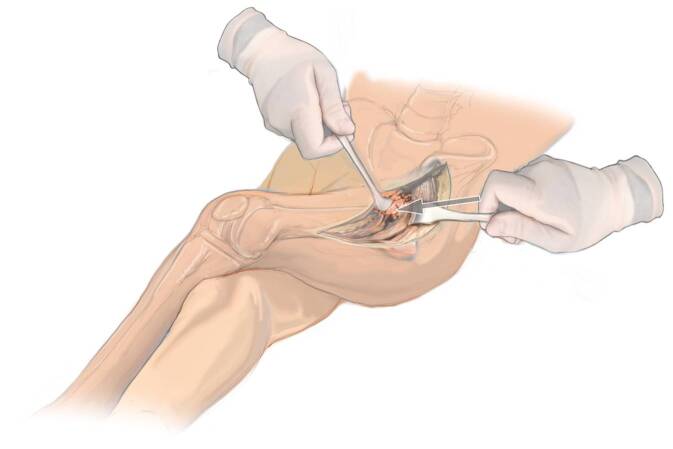
This is a view of a direct anterior proximal femoral replacement looking superiorly from the foot of the table. The left operative hip has been adducted in a scissor fashion over top of the right leg. This “books” or “shotguns” the introitus of the femoral canal out of the wound for in-line access to the femoral shaft. More adduction of the operative leg is possible in the supine position than in the lateral position

**Fig. 8:**
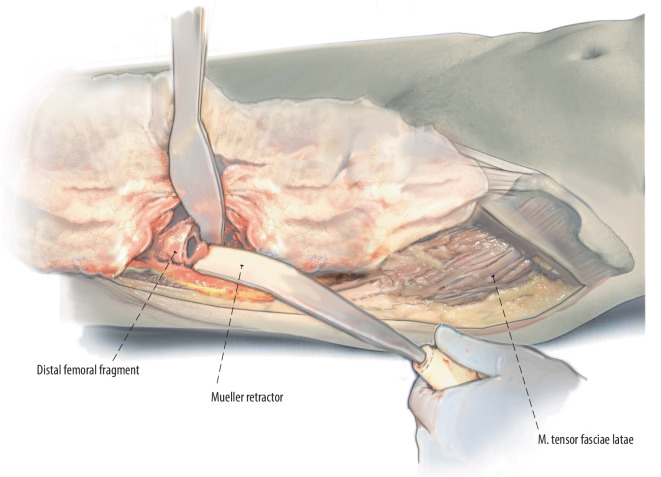
A Mueller retractor is placed around the posterior aspect of the femoral introitus and cobra retractor medial to it. The leg is adducted and crossed over the nonoperative leg. This allows for straight access to the femur. Cerclage wires or beaded cables can used around the introitus of the femur avoid intraoperative fractures at the femur

**Fig. 9:**
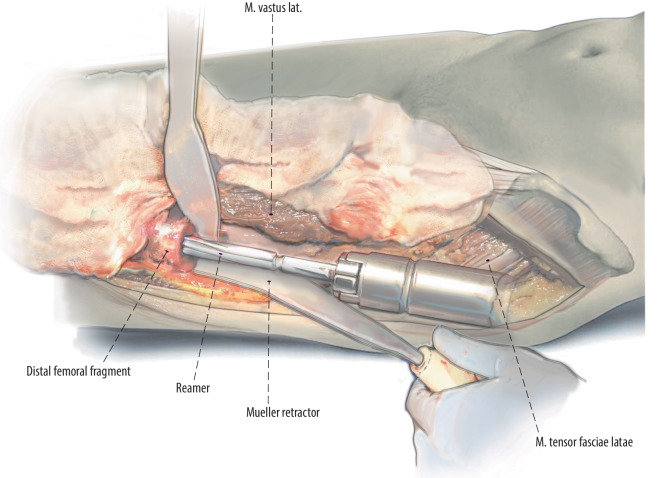
Preparation of the femur with a straight or flexible reamer. The linea aspera and other clinical cues are used to gauge component rotation. Some surgeons prefer to mark femoral rotation with a cautery or an osteotome

**Fig. 10:**
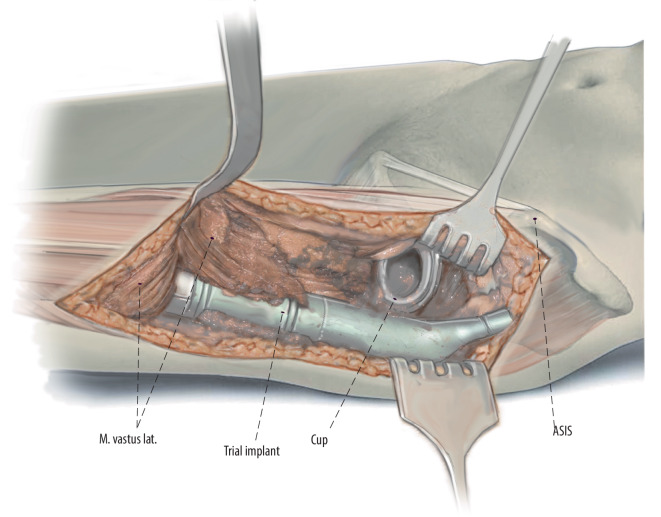
With trial femoral implants in place, careful assessment of implant rotation is undertaken. With the femoral neck positioned in 15°of anteversion the second metatarsal should point directly towards the operating room ceiling. As a second check, the linea aspera can be used if still present. As a third check, we assess for impingement with deep flexion and internal rotation as well as external rotation and extension of the hip. In most patients undergoing proximal femoral replacement, the landmarks used to template and intraoperatively judge leg length restoration are absent. One of the advantages of the anterior approach is that, if both legs are draped, the heels and medial malleoli can be used to compare leg length

**Fig. 11:**
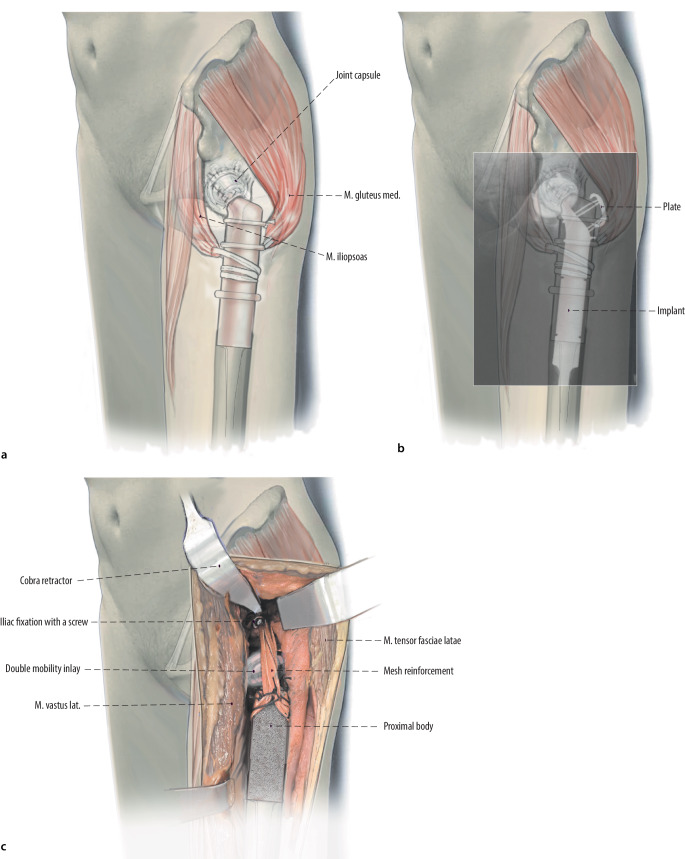
**a** Repair of the abductors back down to the proximal body of the proximal femoral replacement using #5 Ethibond. Therefore, the gluteus medius muscle and the psoas muscle are attached the lateral and medial aspects of the prosthesis. The Ethibond sutures are run through the muscles or the tendons of the muscles and the predefined holes at the implant site. **b** If the greater trochanteric fragment is present, two to four monofilament wires, with different techniques of tunnels in a vertical and horizontal plane through the osteotomized fragment can be used. Claw plates, cables or standard plates can also be used for fixation of the fragment on the prosthesis. The trochanteric fragment should be reattached to the proximal femoral replacement implant at the conclusion of the procedure. Most modern proximal femoral implants have porous ingrowth surfaces just for this purpose around the proximal aspect of the implants to increase the potential of bony ingrowth. In general, the use of dual mobility components on the acetabular side is also highly recommended to avoid dislocations. **c** In select cases of profound instability, we have fortified the connection between the proximal femoral replacement and the acetabulum using a mesh reinforcement. Either an 8 mm Dacron vascular graft or mesh can been looped around the proximal femoral replacement body and secured using #5 Ethibond. The other end of the Dacron vascular graft is secured to the ilium proximal to the acetabular component using 4.5 mm cortical large fragment screws with washers. Usually, a separate screw is used for each Dacron limb

**Fig. 12:**
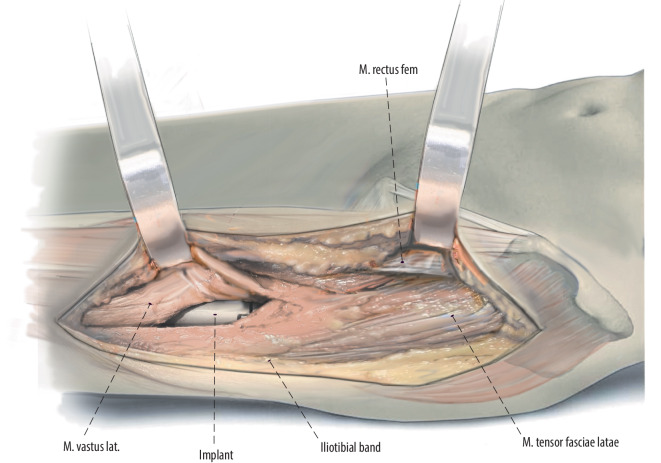
During closure, the vastus lateralis muscle is restored over top of the final components. The posterior border of the vastus can be sutured to the iliotibial band in this area. Usually, the iliotibial band is closed by interrupted 0 PDS sutures and the fascia over the tensor fascia lata using a running 0 PDS suture. A barbed suture and glue-strip dressing is used for subcutaneous and skin closure, respectively

## Special surgical considerations

Figure 13.

**Fig. 13:**
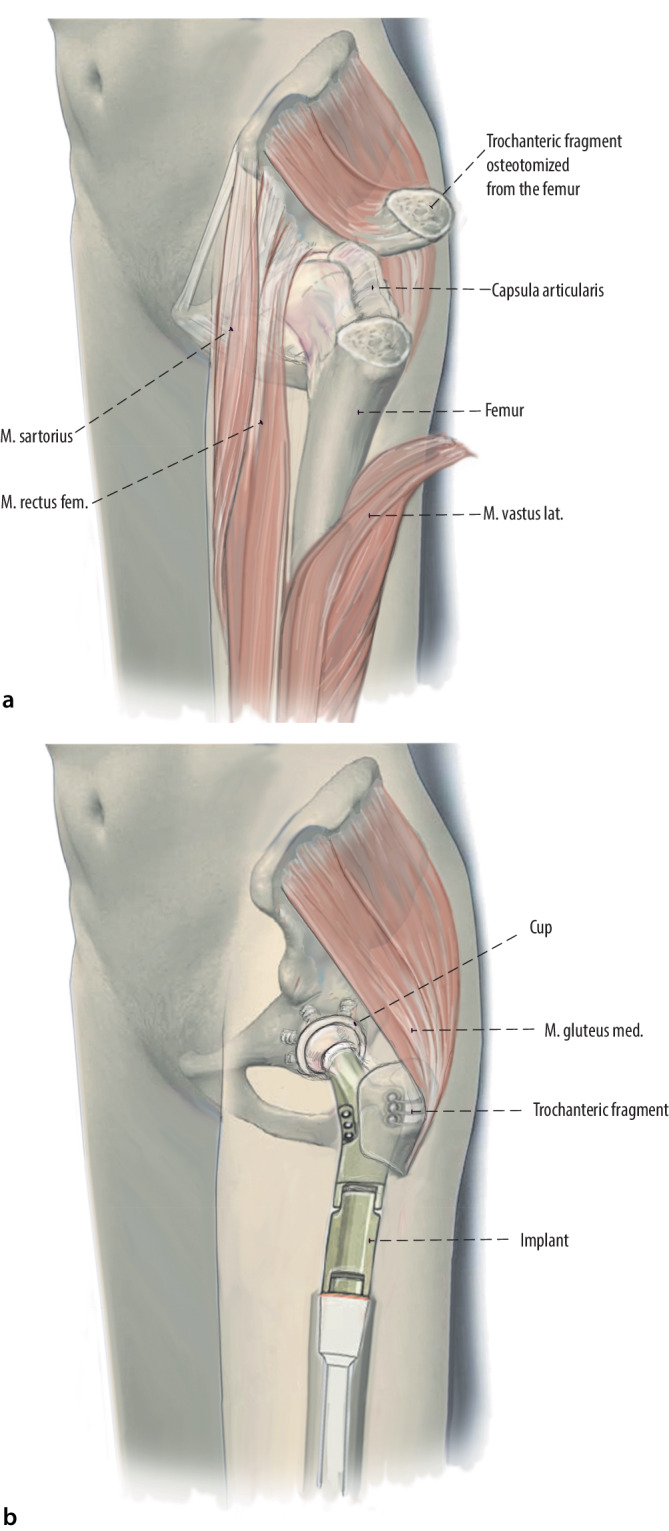
Abductors repair is crucial for the outcome of the patients to avoid postoperative limp and pain. **a** Therefore, as an alternative method, a trochanteric osteotomy can be performed. The trochanteric fragment can be mounted on the proximal femur replacement (PFR) prosthesis during reconstruction. Therefore, no muscles have to be detached because the origin of abductor and vastus lateralis muscle is located posteriorly and laterally. A blunt retractor is then inserted behind the greater trochanter. An oscillating saw or an osteotome is used to make the osteotomy from anterior to posterior, at an angle less than 45° to the femoral diaphysis. In oncological cases this method can only be performed if the trochanteric osteotomy still provides tumor-free margins. Otherwise, this technique is absolutely contraindicated. The osteotomy is then freed of tethering soft tissue attachments and the fragment can be reflected laterally or proximally. **b** A successful fixation technique must provide compression at the osteotomy site and resist both proximal displacement and anterior-posterior (AP) rotation due to the pull of the abductors. Two to four monofilament wires, with different techniques of tunnels in a vertical and horizontal plane through the osteotomized fragment can be used. Claw plates, cables or standard plates can also be used for fixation of the fragment on the prosthesis. If the vastogluteal sling can be protected during surgery, the patient’s functional results might be better

## Postoperative management

- We usually allow full weightbearing immediately after surgery.
- Acetabular reconstruction may limit the patient’s weightbearing postoperatively.
- Limiting combined external rotation and extension for 6 weeks.
- Abduction braces are only utilized in cases of profound soft tissue laxity. This rare postoperative treatment might be indicated in oncological cases with extended resection of musculature.

## Errors, hazards, complications

- Postoperative deep wound infection: Aggressive treatment of early wound healing difficulties and a low threshold to exchange all modular components are good practice to prevent long-term difficulties.
- Dislocation: use of dual mobility bearings should be considered, especially in cases of abductor deficiency.
- Periprosthetic fracture
- Subsidence

## Results

PFR was performed in 16 patients (mean age: 55.1 years; range 17–84 years) using an extension of the DAA. The indication was a primary bone sarcoma in 7 patients. Three patients had a chondrosarcoma and 4 patients an osteosarcoma of the proximal femur. Six patients had a metastatic lesion of the proximal femur. Three patients suffered from massive periprosthetic femoral bone loss of the proximal femur (Paprosky IIIB and IV). Two patients had a dislocation after the surgery; both were treated by closed reduction. One of the 2 patients required reoperation due to recurrent dislocation. Six months after the index surgery a cup revision with the implantation of a double mobility construct was performed. One patient died because of his disease (osteosarcoma). Mean follow-up time was 34.5 months. There were no cases of revision for periprosthetic fracture, mechanical loosening, implant fracture, or subsidence.

## Declarations

All procedures followed were in accordance with the ethical standards of the responsible committee on human experimentation (institutional and national) and with the Helsinki Declaration of 1975 (in its most recently amended version). Informed consent was obtained from all patients included in the study.
